# Food- and Waterborne Bacteria Transmission and Antimicrobial Resistance Under Climate Change: Evidence Gaps in Mainland Portugal

**DOI:** 10.3390/microorganisms14102231

**Published:** 2026-10-03

**Authors:** Mariana Borges, Angela Pista, Leonor Silveira

**Affiliations:** National Reference Laboratory for Gastrointestinal Infections, Department of Infectious Diseases, National Institute of Health Doutor Ricardo Jorge, Avenue Padre Cruz, 1649-016 Lisbon, Portugal; mariana.borges@insa.min-saude.pt

**Keywords:** one health, pathogen dissemination, environmental surveillance, extreme weather events, *Salmonella* spp., *E. coli*, *Vibrio* spp., Enterobacterales

## Abstract

Climate change is increasingly recognised as a driver of microbial risks and antimicrobial resistance (AMR) dynamics across environmental and human systems. This review synthesises current evidence on how climate-related factors may affect the persistence, dissemination, and AMR profiles of some food- and waterborne bacterial pathogens, using mainland Portugal as a climate-vulnerable Mediterranean case study. The analyses focused on *Salmonella* spp., pathogenic *Escherichia coli*, *Vibrio* spp., *Listeria monocytogenes*, *Shigella* spp. and *Yersinia enterocolitica*, integrating findings from environmental, food-chain, and clinical research within a One Health framework. Evidence associates increasing temperatures with enhanced bacterial growth and shifts in seasonal dynamics. Extreme rainfall and flooding events promote faecal contamination through runoff and sediment resuspension, compromising water quality. Drought conditions may concentrate microbial loads and promote the use of alternative water sources, introducing additional exposure pathways. Aquatic and terrestrial systems emerge as critical interfaces for pathogen transmission and AMR dissemination. In mainland Portugal, data from clinical, food, animal, wildlife, and environmental studies are available. However, studies linking climate variables with environmental metadata, AMR and genomic data remain limited. Climate-informed One Health surveillance, through longitudinal sampling, harmonised metadata, whole-genome sequencing and predictive modelling, is essential to anticipate and mitigate climate-driven microbial and AMR risk.

## 1. Introduction

Climate change has been shown to alter climate variability and the frequency and intensity of extreme weather events [1]. Climate change is also increasingly associated with infectious disease risk since it can modify environmental conditions that may influence pathogen survival, replication, dispersal and human exposure [2]. For food- and waterborne (FW) pathogens, these effects are particularly relevant as transmission often depends on climate-sensitive interfaces, such as food systems, surface waters, wastewater, soils, sediments, estuaries and coastal ecosystems [3,4,5].

Food- and waterborne diseases (FWDs) remain a significant global public health burden and an obstacle to socio-economic development [6,7]. According to the World Health Organization (WHO), an estimated 866 million illnesses and 1.52 million deaths worldwide were attributable to the consumption of contaminated food in 2021 [8]. Recent data from the European Union (EU) highlighted an increasing five-year trend in several zoonoses, with 6558 foodborne outbreaks reported, resulting in 62,481 human cases and 53 deaths [9]. Among bacterial pathogens, *Salmonella* spp., pathogenic *Escherichia coli* (including Shiga toxin–producing *E. coli*, STEC), *Shigella* spp., *Vibrio* spp., *Listeria monocytogenes* and *Yersinia enterocolitica* are particularly relevant due to their complex transmission pathways through food, water, animals, humans and contaminated environmental matrices [6,7,9,10].

Climate-related changes can affect these pathogens through several mechanisms. Rising temperatures may increase bacterial growth in food products or aquatic environments and influence the seasonality of infections [4,11,12]. Extreme rainfall and flooding can increase faecal contamination of surface waters through runoff, sewage overflow, wastewater-treatment disruption and sediment resuspension [13,14,15]. Drought and water scarcity may increase reliance on alternative water sources, including reclaimed wastewater for irrigation [16].

Antimicrobial resistance (AMR) adds a further dimension to this problem. Inadequate antimicrobial use in humans, animals and agriculture remains the principal driver of resistance, but the environment is increasingly recognised as a reservoir and part of the dissemination pathways for antimicrobial-resistant bacteria (ARB) and -antimicrobial resistance genes (ARGs) [17,18,19]. Wastewater treatment plants, surface waters, estuaries, agricultural soils and sediments may facilitate the persistence and spread of resistance determinants across human, animal and environmental interfaces [17,18]. Although climate change should not be presented as the primary cause of AMR, climate-related changes may modify the environmental dynamics and dissemination pathways of AMR. Flooding and extreme rainfall can promote the mobilisation and transport of resistant bacteria and ARGs from wastewater systems, agricultural land and contaminated soils into surface waters, while rising temperatures may influence bacterial growth, survival and the dynamics of resistance in environmental reservoirs. Conversely, drought and water scarcity may increase reliance on alternative or lower-quality water sources and concentrate contaminants, potentially increasing opportunities for exposure to ARB [19,20]. These interactions highlight the need to consider AMR within a One Health framework when assessing the effects of climate change on FW pathogens.

Mainland Portugal provides a relevant case study for investigating these interactions within a One Health framework. As part of the Mediterranean-climate region, it is increasingly exposed to climate-related pressures, including rising temperatures, heatwaves, droughts, water scarcity, extreme rainfall, and coastal events [3,21]. These environmental changes are particularly relevant to microbial risk since the country encompasses extensive coastal and estuarine areas, major shellfish production areas, agricultural regions dependent on irrigation, and increasing interest in water reuse as an adaptation strategy to address drought and water scarcity [16,21].

Despite this vulnerability, the Portuguese evidence base remains fragmented. Studies have investigated AMR in clinical, animal, food, and some environmental settings, particularly for *E. coli*, but relatively few have integrated climate variables, environmental matrices, FW pathogens and AMR within a single One Health framework [19,22,23]. This gap limits understanding of how climate conditions may influence pathogen persistence and transmission, as well as the dissemination of AMR across human, animal, food and environmental interfaces in mainland Portugal.

This literature review synthesises available evidence on how climate-related factors may influence the persistence, dissemination, and AMR profiles of selected FW bacterial pathogens, using mainland Portugal as a climate-vulnerable Mediterranean case study. The review focuses only on pathogens within the authors’ established areas of expertise and ongoing research, including *Salmonella* spp., *E. coli*, *Shigella* spp., *Vibrio* spp., *L. monocytogenes*, and *Y. enterocolitica*. A One Health perspective is applied to explore the interplay between climatic pressures, pathogen ecology and dynamics, and the circulation of ARB and resistance determinants across human, animal, food, and environmental interfaces.

Literature for this review was identified through a series of targeted PubMed searches, selected for its broad coverage of biomedical and microbiological literature relevant to FW bacterial pathogens, transmission and AMR, as well as its free accessibility as a bibliographic database. Searches were structured according to each pathogen and thematic area. Search strategies combined the six bacterial pathogens of interest (*Salmonella* spp., pathogenic *E. coli*, *Vibrio* spp., *L. monocytogenes*, *Shigella* spp. and *Y. enterocolitica*) with terms related to climate and transmission, AMR, and Portugal. Additional searches were conducted to provide broader international context, and citation tracking of key review articles and outbreak reports was used to identify further relevant studies. International reviews were used to establish the general climate–pathogen–AMR framework supporting each section, and primary research articles were subsequently selected to substantiate specific mechanisms or to provide relevant empirical or comparative evidence. The search strategy, publication period, inclusion and exclusion criteria, and study selection process are described in Supplementary Data S1.

Given the broad range of pathogens, environmental compartments and thematic areas addressed, findings were integrated through a narrative synthesis within a One Health framework rather than through a formal systematic review.

## 2. Environmental Interfaces of Pathogen Transmission and Antimicrobial Resistance

Environmental compartments act as dynamic interfaces in the ecology of FW bacterial pathogens, influencing their occurrence, persistence, dispersal and exposure potential [24,25]. Freshwater systems, wastewater networks, agricultural soils, sediments and estuarine and coastal ecosystems differ substantially in physicochemical conditions, microbial communities and anthropogenic pressures, resulting in distinct roles in pathogen transmission and AMR dissemination [26].

Freshwater systems receive water inputs from multiple sources, including wastewater discharges, livestock production, wildlife and agricultural runoff, creating important pathways for the dissemination of enteric bacteria across catchments [26]. Rivers and reservoirs can connect rural, urban and coastal environments, facilitating the faecal contamination of irrigation systems, recreational waters or shellfish-harvesting areas [14,26]. These pathways are particularly relevant for pathogens, such as *Salmonella* spp., pathogenic *E. coli*, *Shigella* spp., and *L. monocytogenes*, which may contaminate fresh produce when microbiologically unsafe water is used for irrigation before harvest [27].

Wastewater systems represent one of the major One Health interfaces for AMR, as they concentrate microorganisms, antimicrobial residues and ARGs from households, healthcare facilities, food-processing industries and other anthropogenic activities [18]. Although treatment processes reduce microbial loads, treated effluents may still release ARB and ARGs into receiving aquatic environments [18,28]. Large-scale metagenomic studies of urban sewage have demonstrated that wastewater can provide valuable insights into global AMR patterns, highlighting its potential as a matrix for environmental surveillance [28,29].

Agricultural soils are important reservoirs of enteric bacteria and resistance determinants due to inputs from manure, slurry, sewage sludge, contaminated irrigation water and wildlife activity [24,27]. The persistence and survival of bacterial pathogens in soil are influenced by multiple physicochemical and ecological factors, including moisture content, temperature, organic matter availability, pH, soil structure and interactions with local microbial communities [25,30]. Soil contamination is of particular concern for fresh produce safety, as pathogens introduced before harvest may reach edible plant surfaces directly or indirectly through splash dispersal, irrigation or contact with contaminated soil particles [24,27].

Sediments can act as secondary reservoirs for faecal bacteria, as bacterial cells may attach to particles, form biofilms, and persist for longer periods than in the overlying water column [15,25]. Hydrological disturbances can resuspend sediment-associated bacteria and reintroduce them into surface waters, generating episodic contamination events that may be overlooked by routine water-column sampling [15,25]. Sediment-associated microbial reservoirs should therefore be considered in water-quality assessments, as sediment disturbance can temporarily increase microbial loads in the water column [15].

Estuarine and coastal ecosystems integrate microbial inputs from rivers, wastewater discharges, agricultural runoff and tidal processes [25,26]. These environments are highly relevant to food safety since they support aquaculture, shellfish harvesting and recreational activities, which represent direct exposure pathways for humans [31,32]. In addition, brackish waters provide suitable habitats for naturally occurring marine pathogens, such as *Vibrio* spp., whose abundance and distribution are strongly influenced by sea surface temperature, salinity and other coastal environmental conditions [31,33]. Changes in these conditions, particularly increasing water temperatures, may therefore affect the occurrence and geographic distribution of *Vibrio* spp. and associated human health risks.

In mainland Portugal, environmental and wildlife-associated reservoirs of FW bacteria and AMR have been studied, although evidence remains uneven across pathogens and matrices. Studies of wildlife and free-living birds have detected pathogenic *E. coli*, and *Salmonella* spp. in mainland Portugal, using phenotypic characterisation, PCR and whole-genome sequencing (WGS), indicating that wild animals and birds may act as reservoirs of zoonotic bacteria and AMR strains [22,23]. Studies of urban birds in Lisbon have also identified extended-spectrum beta-lactamases (ESBL)-producing *E. coli* in pigeons and high rates of ESBL- and carbapenemase-producing Enterobacterales in gull faeces from the Lisbon coastline, suggesting that urban and coastal birds can reflect anthropogenic contamination and contribute to environmental AMR dissemination [34,35]. In aquatic settings, a Portuguese study has reported the occurrence of pathogenic and AMR *E. coli* in recreational waters, indicating that human exposure to resistant enteric bacteria may occur through environmental water contact [36]. However, integrated assessments combining climate variables, environmental matrices, pathogen occurrence and AMR remain scarce. Evidence is particularly limited regarding the influence of climate conditions on *E. coli*, *Salmonella* spp., *Shigella* spp., *Vibrio* spp., *L. monocytogenes* and *Y. enterocolitica* across Portuguese freshwater, soil, wastewater, sediment, estuarine and coastal systems.

Importantly, these reservoirs are not static and may be altered by changes in environmental conditions driven by climate change (Figure 1). Temperature, precipitation patterns, droughts and extreme hydrological events can modify water availability, nutrient dynamics, sediment transport, salinity gradients and microbial community composition, thereby affecting the persistence, distribution and dispersal of both pathogens and AMR determinants [3,14,37,38]. Increased rainfall and flooding may enhance the spread of microorganisms between soils, wastewater systems and surface waters, whereas drought conditions can reduce dilution capacity and increase the concentration of microbial contaminants in aquatic environments [13,37]. Similarly, rising temperatures may influence microbial survival and ecological interactions in freshwater, soil, estuarine and coastal ecosystems, potentially altering the pathways through which FW pathogens are maintained and transmitted [39,40]. Therefore, understanding these interactions is essential for anticipating how climate change may shape future risks associated with pathogen transmission and AMR dissemination within a One Health framework.

## 3. Mainland Portugal as a Climate-Sensitive Case Study

Mainland Portugal occupies the western margin of the Iberian Peninsula and has an extensive Atlantic coastline, although its climate its predominantly Mediterranean with strong Atlantic and coastal influences, reflecting its position as a transitional zone between subtropical and temperate climates [41]. The northwestern and mountainous areas are generally cooler and wetter, whereas the interior and southern regions experience higher temperatures, lower annual precipitation and longer summer dry periods [41]. Several major river basins, including the Minho, Douro, Tagus and Guadiana, are shared with Spain, making water availability and quality partly dependent on transboundary river management [42].

Water availability varies across the country, where drought and water scarcity occur most frequently and severely in the south, particularly in the Alentejo and Algarve, although prolonged dry conditions can affect most of mainland Portugal [21]. The Portuguese Environment Agency estimated a national water-scarcity index of 34% for 1989–2015, with substantially higher values in the Sado and Mira (74%) and Algarve (66%) river basins [43]. Climate projections for mainland Portugal indicate a continued decline in annual precipitation across the 21st century, with decreases of up to 20% by 2041–2070 and up to 40% by 2071–2100 in southwestern Portugal under a high-emissions scenario, with the Algarve region projected to be particularly affected. The frequency and duration of moderate droughts are also projected to increase substantially [44]. Agriculture accounts for approximately 70% of national water consumption, making irrigation particularly vulnerable to reduced water availability [43].

Beyond drought, intense precipitation and flooding also directly threaten Portuguese socio-economic activities, including agriculture, with 154 flood events recorded during the 2023/2024 hydrological year causing major impacts across vulnerable river basins [21]. Aquaculture is particularly exposed since 97.8% of national production originated from marine and brackish waters in 2022, and was concentrated in coastal lagoons and estuarine systems, including Ria de Aveiro, the Mondego, Tagus and Sado estuaries and Ria Formosa [21].

Portugal has achieved extensive urban wastewater collection and treatment coverage, although differences in infrastructure performance and service uptake remain. In 2022, 57% of public wastewater treatment plants serving agglomerations of at least 2000 population equivalents provided secondary treatment and 42% provided more advanced treatment, with 95% compliance based on the treated load [21]. Water reuse is being promoted as an adaptation to drought and water scarcity under a risk-based regulatory framework, particularly for agricultural irrigation, making the microbiological quality of reclaimed water and receiving environments increasingly important surveillance targets, since treated effluents may remain relevant pathways for ARB and ARGs [16,21,45].

These geographic, climatic, land use and water-management characteristics make mainland Portugal a relevant Mediterranean case study for FW bacterial pathogens, and also demonstrate that exposure is unlikely to be spatially uniform, supporting surveillance approaches that account for regional climate, hydrological events, land use, irrigation, water reuse, wastewater discharges, aquaculture and recreational water use.

## 4. Food- and Waterborne Bacterial Pathogens: Influence of Climate Factors on Transmission, Persistence and Antimicrobial Resistance

The following subsections assessed each bacterial pathogen by integrating the Portuguese evidence base and synthesizing international evidence on the relevant climate-sensitive pathways. The selection was targeted rather than exhaustive, since these pathogens were chosen for their clinical and epidemiological relevance, transmission pathways and environmental niches, potential relevance to climate-sensitive pathways in mainland Portugal, and alignment with the laboratory expertise.

All six pathogens are subject to routine epidemiological surveillance and reporting across the EU, given their recognised public health importance as major causes of FWD [9]. *Salmonella* spp. are major zoonotic foodborne pathogens responsible for the second most frequently reported human gastrointestinal infection in the EU, predominantly causing foodborne illness and outbreaks that carry a significant burden of hospitalisations [9]. Pathogenic *E. coli* comprise several pathotypes associated with diarrhoeal disease, while STEC can result in severe clinical outcomes, including bloody diarrhoea and haemolytic uraemic syndrome [9]. *Vibrio* spp. are primarily associated with marine and estuarine environments and can cause seafood-associated gastroenteritis, wound infections, septicaemia and, in the case of toxigenic *V. cholerae*, cholera [31]. *L. monocytogenes* causes severe invasive disease, particularly among pregnant women, neonates, older adults and immunocompromised individuals, with invasive listeriosis classified as the most severe zoonotic disease monitored across EU Member States [9]. *Shigella* spp. are human pathogens with a low infectious dose that can cause gastrointestinal disease, including diarrhoea and dysentery, with transmission closely related to water, sanitation and hygiene conditions [46]. *Y. enterocolitica* is a zoonotic bacterial pathogen frequently detected in pigs and fresh pork meat and is responsible for human yersiniosis and foodborne outbreaks across the EU [9].

Although Portugal has generated substantial research on some of these pathogens across clinical, food-chain, animal, wildlife and environmental settings, including AMR, studies explicitly assessing how climate variables and extreme weather events influence their transmission, persistence or AMR in mainland Portugal remains scarce (Supplementary Table S1).

In Portugal, mandatory notification through the national epidemiological surveillance system includes typhoid and paratyphoid fever, non-Typhi and non-Paratyphi salmonellosis, STEC infection including haemolytic uraemic syndrome, toxigenic *V. cholerae*, listeriosis, shigellosis, and yersiniosis [47].

### 4.1. Salmonella *spp.*

*Salmonella* spp. are among the FW pathogens most consistently associated with temperature-sensitive transmission. European and international studies have reported positive associations between ambient temperature and salmonellosis incidence, although the magnitude of this effect varies according to food-chain practices, surveillance systems and local environmental conditions [11,12,48,49]. Recent reviews also highlight that temperature may affect *Salmonella* spp. at multiple stages of the food chain, including animal production, transport, storage and food-processing environments [50,51].

Environmental reservoirs are also relevant since *Salmonella* spp. can persist outside hosts in water, soils, sediments, manure and low-moisture environments, creating multiple opportunities for contamination before harvest or during food processing [24,52,53]. Heavy rainfall and flooding may facilitate contamination from livestock areas, manure-amended soils, wastewater systems and contaminated sediments into surface waters and agricultural environments, while contaminated irrigation water may facilitate the dissemination of *Salmonella* spp. to fresh produce [27,39]. These mechanisms are particularly relevant under climate-change scenarios characterised by increasing hydrological extremes.

AMR in *Salmonella* spp. is primarily driven by antimicrobial use in human and animal sectors, although environmental dissemination may contribute to the spread of resistant strains and ARGs through wastewater, runoff, manure and contaminated water systems [45].

In Portugal, *Salmonella* spp. has been extensively studied across clinical, food-chain, animal, wildlife and environmental matrices, including outbreak investigations, clinical samples, livestock, poultry and pig production, slaughterhouses, food products, wildlife, pets, aquaculture, recreational waters, and coastal and estuarine waters, generating one of the most substantial national evidence bases among the pathogens covered in this review (Supplementary Table S1) [22,23,54,55,56,57,58,59,60,61,62,63,64,65,66,67,68,69,70,71,72,73,74,75,76,77,78,79,80,81]. However, none of these studies explicitly examines temperature, rainfall, flooding, drought or other climate-related effects as factors influencing *Salmonella* spp. occurrence or persistence, or the environmental dissemination of ARB and ARGs, leaving this specific climate dimension unaddressed.

International evidence provides increasingly quantitative support for associations between climatic conditions and *Salmonella* spp. transmission. An analysis of 275,753 Australian salmonellosis notifications over a 29-year period identified distinct seasonal fluctuations, with notifications peaking during the summer and dropping to lower levels in winter. The study estimated a 3.4% increase in cases per 1 °C rise in monthly mean temperature anomaly, with a further 7.9% increase during El Niño periods [82]. A comparative risk-assessment model attributed 11% of the national *Salmonella* disease burden between 2003 and 2018 to non-optimal high temperatures, with the greatest attributable burden occurring in tropical areas. This burden was projected to increase substantially by the 2050s under a high-emissions scenario [83].

In the United States (US), FoodNet surveillance data from 2004–2014 showed that extreme-heat days were associated with a 6–7% increase in *S*. Newport infection rates in states characterized by dense animal feeding operations, such as poultry and livestock production [84]. A separate ecological analysis of the Southwest US (2009–2021) found that each additional inch of precipitation was associated with a 2% increase in *Salmonella* cases two weeks later. This association was amplified to 16–22% following moderate-to-severe antecedent drought, whereas case numbers decreased under the most extreme drought conditions [85].

At the European scale, a landmark review co-authored by researchers from the ECDC and WHO Europe highlighted that weekly ambient temperatures above 5 °C were associated with a 5–10% increase in salmonellosis notifications for each 1 °C rise. The study further estimated that temperature-related effects could account for approximately one-third of salmonellosis transmission in England and Wales, Poland, the Netherlands, the Czech Republic, Switzerland, and Spain [86]. Seasonal effects have also been quantified in Greece, where national retail poultry surveillance reported *Salmonella* spp. isolation rates of 60.4% in summer carcasses compared with 18.7% in winter [87].

The climate–AMR relationship for *Salmonella* spp. has also begun to be quantified. A five-year-scale ecological study in China found that each 1 °C increase in temperature variability was associated with an 8% rise in ARGs prevalence in southern China, while an inverse association was observed in the north, with a 10% decrease [88].

An analysis of 488,232 *Salmonella* genomes from 139 countries or regions, spanning 1940–2023, estimated that climate change was associated with a 10% global increase in ARG abundance, with increases observed in 82 of the 100 countries analysed, independently of traditional socioeconomic factors and antimicrobial selection pressures [2].

Regarding extreme weather events, following Hurricane Florence in 2018, environmental monitoring in North Carolina, USA, detected persistent, AMR *Salmonella enterica* lineages in flood-affected surface waters. Rather than being seeded by the storm or upstream agricultural facilities, high-resolution genomic sequencing revealed these pathogens originated from a stable, pre-existing environmental reservoir [89]. In India, after the devastating 2018 Kerala flood (the region’s worst flooding event in nearly a century) metagenomic characterization of the post-flood bacterial resistome (including *Salmonella* spp.) in affected agricultural sediments identified substantial ARGs carriage, including 103 unique genes. Pathogenic strains persisted in the environment and remained detectable in follow-up samples collected six months after [90]. A similarly severe flood in a Chinese man-made river system was linked to both *Salmonella* spp. persistence and elevated AMR in the resulting isolates [91]. A study following the catastrophic October 2024 DANA flash flood in Valencia, Spain, found viable opportunistic pathogens from genus *Salmonella* and *Shigella* among others detected by qPCR in enriched flood-derived sludge collected one month after the event, alongside isolated *E. coli* and other Enterobacterales [92]. A parallel study of the adjacent Albufera Natural Park wetland found the flood altered water chemistry and pathogenic bacteria levels, with the most heavily contaminated areas linked to wastewater treatment failures and urban runoff [93].

Despite Portugal’s recent experience of severe storms and flooding, no published studies were identified that have used such events as a case studies to investigate *Salmonella* spp. contamination, environmental persistence, transmission or outbreak risk, representing a missed opportunity to generate valuable evidence.

Overall, the international evidence highlights a significant and addressable knowledge gap in Portugal, where the influence of climatic factors on *Salmonella* spp. occurrence, transmission and AMR remains poorly studied despite the extensive national evidence base on this pathogen. Future studies should therefore integrate environmental sampling, antimicrobial susceptibility testing, WGS and high-resolution climate and meteorological data to investigate these relationships within a One Health framework.

### 4.2. Pathogenic Escherichia coli

Pathogenic *E. coli* is relevant to climate-sensitive FWDs since, beyond the use of generic *E. coli* as a standard faecal indicator, several pathotypes, particularly STEC, are associated with animal reservoirs, fresh produce, water exposure and food-chain contamination [9,94,95]. Climate-related factors, such as higher temperatures and heavy rainfall events, have the potential to boost the survival and occurrence of disease-associated bacteria in the environment and to facilitate their dispersal from farms and sewage systems into river catchments, influencing these pathways indirectly by altering rainfall–runoff dynamics, faecal contamination of agricultural water, irrigation practices and the persistence of enteric bacteria in food-production environments [14,27]. Warmer temperatures and altered rainfall can affect the persistence and distribution of *E. coli* on crops, while water scarcity may increase reliance on untreated or inadequately treated wastewater for irrigation, creating opportunities for the introduction of pathogenic *E. coli* into the food supply chain [51]. Recent reviews and outbreak investigations further indicate that heatwaves, altered precipitation and water scarcity may affect STEC exposure through enhanced livestock shedding, crop contamination and the use of inadequately treated water in agriculture [50,51].

In Portugal, *E. coli* has been studied across several non-climate-specific contexts, including food safety, food-producing animals, wildlife, companion animals, aquatic environments and clinical and One Health surveillance, including AMR (Supplementary Table S1) [22,23,34,35,36,61,69,74,80,81,96,97,98,99,100,101,102,103,104,105,106,107,108,109]. In the Ria Formosa coastal lagoon, monitoring of faecal indicators across three zones over two years found the highest contamination level near the highest-load, least-flushed discharge point. Hydrodynamics, temperature, rainfall, land runoff, and seabird populations all contributed to contamination levels in adjacent bivalve production areas. A complementary bivalve-specific water quality index applied to the same lagoon system showed that water quality improved with increasing distance from effluent discharge points and during colder months, whereas poor water quality under strong hydrodynamic conditions was associated with warm-season toxigenic phytoplankton [110,111]. However, none of these Portuguese studies explicitly analyses climate variables and extreme weather events as drivers of *E. coli* transmission, environmental persistence or AMR, leaving this specific climate dimension unaddressed nationally.

Internationally, this link has been more extensively studied and quantified. A landmark example is the 2022 STEC O157:H7 outbreak in the UK, in which heavy rainfall and flooding were implicated in the contamination of lettuce crops with STEC from animal faeces, with a geospatial One Health analysis triangulating meteorological, flood-risk, animal-density and land-use data used to identify the contamination pathway [112]. A similar flood-associated STEC outbreak was documented in Belgium following the mid-July 2021 floods, involving ten O26:H11 cases who all developed haemolytic uremic syndrome. WGS confirmed a core cluster comprising six of these cases, and epidemiological investigation linked the outbreak to the consumption of contaminated tap water in the severely flooded province of Belgian-Luxembourg [113].

Precipitation effects have also been quantified in urban coastal systems. Monitoring storm drain outfalls in Tampa Bay, Florida, revealed that average concentrations of culturable *E. coli* and *Enterococcus* had a ten-fold rise following wet weather compared to dry baselines. Furthermore, detection of the *E. coli* virulence gene *eae* increased during wet periods, and its abundance was significantly correlated with 20 distinct ARGs [114]. Similarly, in the River Trent, England, upstream *E. coli* concentrations increased from a baseline of 413 CFU/100 mL to 14,843 CFU/100 mL, a 36-fold increase, following heavy rainfall and upstream sewer overflow discharges [115].

Flood-specific dynamics have also been documented in floodplain ecosystems. A study conducted in an Eastern France floodplain found that *E. coli* (including ESBL-producing strains), *Klebsiella pneumoniae*, and *Pseudomonas aeruginosa* were dispersed through aquatic networks predominantly during high-flow hydrological periods, with most *E. coli* isolates likely originating from human sources [116]. Post-flood monitoring of an Australian metropolitan river revealed that 40.6% of the *E. coli* isolates shared identical biochemical fingerprints with isolates recovered from upstream wastewater treatment plants within the same catchment. Although overall *E. coli* concentrations decreased in the weeks following the disaster, the carriage of virulence genes was significantly higher in samples collected during the final sampling round than in earlier phases [117]. This quantified international evidence demonstrates clear associations between rainfall and flooding, increased environmental *E. coli* contamination, and the dissemination and persistence of potentially pathogenic and antimicrobial-resistant strains, underscoring the meaningful gap in equivalent research in Portugal.

This evidence base also extends to Mediterranean Europe, encompassing both extreme-event and routine seasonal variations. Following the catastrophic October 2024 DANA flash flood in Valencia, Spain, flood-derived sludge collected one month after the event yielded viable *E. coli* and other Enterobacterales. One isolate exhibited combined multidrug resistance and virulence-associated traits, including biofilm formation, suggesting that flood sediments may act as a persistent reservoir of clinically relevant strains rather than a transient contamination event [92]. In the Ebro and Xúquer river basins in Spain, including the Albufera de València wetland, *E. coli* and ESBL-producing *E. coli* were detected in surface water only during summer and sporadically in spring, while overall antimicrobial concentrations in the water peaked during the winter months, potentially reflecting higher seasonal use combined with reduced environmental degradation [118].

Portuguese studies have largely focused on food safety, animal and wildlife reservoirs, environmental contamination, microbiological quality, and AMR surveillance. Despite Portugal’s recent experience of severe storms and flooding, and its Mediterranean environmental systems comparable to those investigated elsewhere, no published Portuguese studies using extreme weather events or systematic seasonal monitoring as a basis for investigating *E. coli* contamination, environmental persistence, or AMR dynamics were found. This represents an important gap in the evidence base and a missed opportunity to generate climate-relevant insights.

### 4.3. Vibrio *spp.*

Among the pathogens considered in this review, *Vibrio* spp. represent one of the clearest examples of climate-sensitive FW bacteria. Unlike other enteric bacteria whose occurrence is mainly linked to faecal contamination, pathogenic *Vibrio* spp. are naturally associated with marine and estuarine ecosystems, and their abundance is strongly influenced by sea surface temperature, salinity and coastal environmental conditions [31,33,119]. In aquatic settings, the prevalence of *Vibrio* spp. is primarily dictated by salt concentration and water temperature. Consequently, as coastal regions warm, seafood may harbour higher levels of these pathogens, particularly in areas with lower salinity or brackish conditions [120]. Recent One Health reviews identify warming waters, rainfall, flooding, salinity shifts and seafood production as key factors connecting climate change with *Vibrio* spp. transmission through coastal and food-chain pathways [51].

The climate signal is particularly well documented for non-cholera *Vibrio* spp., such as *Vibrio parahaemolyticus* and *Vibrio vulnificus*. Bacteria of the *Vibrio* genus are highly responsive to environmental shifts and typically flourish in aquatic settings where temperatures exceed 17 °C. Projections suggest that under the most severe climate models, the geographic range of these bacteria could expand to include an additional 38,000 km of shoreline by the year 2100 [121]. Ocean warming has been linked to increased environmental suitability, longer seasonal windows and geographical expansion of pathogenic *Vibrio* spp. in regions previously considered less favourable for transmission [119,122]. By integrating climate data with demographic and socioeconomic forecasts, researchers have demonstrated that non-cholera *Vibrio* illnesses have spread worldwide over the last few decades, and these pathogens are now appearing in regions formerly deemed unsuitable for their survival. However, this expansion varies by region, as some areas are facing much faster increases than others [122]. In northern Europe, heatwaves and unusually high coastal water temperatures have been associated with increases in *Vibrio*-related wound and soft-tissue infections, illustrating how short-term extreme events may trigger acute public-health risks in coastal populations [33,119]. Surveillance data from the Baltic Sea region document an increase in *Vibrio* infections over time, with sea surface temperature and salinity emerging as critical drivers of *Vibrio* spp. distribution and abundance, reinforcing the need for integrated climate-informed coastal monitoring [123]. Salinity is also central since many pathogenic *Vibrio* spp. proliferate in brackish waters, meaning that river discharge, drought, rainfall and estuarine mixing can modify risk independently of temperature [31,120].

Foodborne transmission occurs mainly through seafood consumption, especially raw or undercooked bivalve molluscs, as filter-feeding shellfish can concentrate *Vibrio* spp. from surrounding waters [31,120]. This makes *Vibrio* spp. highly relevant for countries with shellfish harvesting, aquaculture and recreational coastal activities. AMR is less well characterised than for *E. coli* or *Salmonella* spp. Nevertheless, resistance determinants have been identified in environmental, aquaculture and clinical *Vibrio* isolates, and EFSA has highlighted AMR in *Vibrio* spp. as an emerging concern in seafood safety under climate change [120,124].

Mainland Portugal may be particularly relevant for climate-sensitive *Vibrio* surveillance since it combines extensive Atlantic coastline with numerous estuarine ecosystems, extensive shellfish production areas and projected increases in sea-surface temperature and marine heatwave frequency [125,126]. Together, these characteristics provide environmental conditions that may favour changes in the ecology, seasonal dynamics and geographical distribution of pathogenic *Vibrio* spp. [120]. In Portugal, *Vibrio* spp. has been studied through outbreak investigation, clinical surveillance, and detection in ready-to-eat shellfish (Supplementary Table S1) [127,128,129,130,131]. Additionally, seasonality was assessed documenting *Vibrio vulnificus* throughout the entire year, including the colder winter months of November, January, and February, while the detection of *Vibrio parahaemolyticus* was limited to the warmer season [130]. Subsequent work in the Tagus estuary documented pronounced seasonal variation in *Vibrio* spp. levels in two commercially harvested clam species and in associated sediments, with significantly higher abundances recorded in summer months, consistent with the temperature-dependence of *Vibrio* spp. ecology described internationally [132]. More recently, a one-year microbiome survey of Pacific oysters (*Crassostrea gigas*), harvested from the Canal de Mira production area in the “Ria de Aveiro” found *Vibrio* spp. to be the dominant bacterial genus during most of the year, with greater dominance outside the winter months, reflecting the expected temperature-driven seasonality of this genus in Atlantic coastal settings [133].

In Portugal, mandatory notification under the national SINAVE system (Despacho n.º 1150/2021) applies only to cholera caused by toxigenic *V. cholerae* O1 or O139 (confirmed by detection of cholera toxin or the cholera toxin gene and one of the specific antigens), while no equivalent surveillance obligation exists for other *Vibrio* spp. or for non-toxigenic *V. cholerae* [47]. Consequently, clinical surveillance is unlikely to provide a comprehensive picture of infections caused by non-cholera *Vibrio* spp., particularly when laboratory testing is not routinely performed or species-level identification is unavailable. The national monitoring programme for Portuguese bivalve production areas provides a systematic framework for tracking microbiological, metal, and biotoxin hazards [125]. Although its microbiological assessment currently focuses on *E. coli* as the primary marker for faecal contamination, the programme has a history of evolving to address consumer safety demands and emerging threats, which suggests it is a viable platform for the future integration of broader surveillance, such as for *Vibrio* spp. [125].

Internationally, temperature and salinity dependence has been demonstrated in the Baltic Sea, where sampling across Finnish, German and Danish coastal sites found *V. vulnificus* occurrence tied specifically to temperature and phosphate concentration, detected only at one site during peak summer months at intermediate salinity [134]. At a broader global scale, a systematic review and meta-analysis of 83 studies covering *Salmonella*, *Shigella*, *Vibrio*, and *Listeria* species confirmed a consistent positive association between ambient temperature and *Vibrio* gastroenteritis. However, the study highlighted that the influence of wet weather is highly region-specific. While precipitation and flooding were positively associated with enteric infections in tropical regions, no such association was observed for *Vibrio* spp. in temperate areas like Europe, where transmission is instead heavily driven by rising sea temperatures [49]. A global One Health systematic review of AMR across 59 countries identified *Vibrio* spp., alongside *Aeromonas* spp. and *Salmonella* spp., as the most prevalent resistant pathogens overall, with resistant strains most frequently isolated from environmental and food sources, particularly aquaculture and seafood [135].

From another perspective, the northwest Iberian Peninsula functions as an interconnected Atlantic coastal environment and the documented emergence of *Vibrio* spp. in Galicia is highly relevant to adjacent territories. In the Galician “rías”, *V. parahaemolyticus* was detected across all seasons, proving its established occurrence in these waters. While salinity is the primary factor regulating the pathogen’s abundance, temperature provides a secondary effect that modulates its abundance. Crucially, research has identified that major changes in the region’s epidemic history coincide with significant warming trends and rising sea surface temperatures [136,137]. A retrospective clinical analysis from southern Spain recorded a sustained increase in *Vibrio*-associated infections over a 13-year period in parallel with the warming trend in coastal waters, providing the most geographically proximate analogue for what may emerge along the Portuguese coast under continued climate warming [138].

Compared to the Mediterranean and European evidence, Portuguese peer-reviewed studies explicitly integrating *Vibrio* spp. occurrence, climate variables and AMR remain scarce. Portugal has established monitoring of shellfish production areas, particularly for microbiological quality and marine biotoxins, but this has not yet translated into a substantial published evidence base on pathogenic *Vibrio* spp. ecology under climate-change scenarios. Evidence concerning AMR in environmental or seafood-associated *Vibrio* spp. isolates from Portuguese coastal matrices is likewise limited. Therefore, *Vibrio* spp. should be framed in the Portuguese context as a priority area for climate-informed coastal surveillance, particularly in estuaries, shellfish production zones and recreational waters.

### 4.4. Listeria monocytogenes

*L. monocytogenes* differs from many enteric foodborne pathogens since it is not primarily linked to faecal contamination, but to broad environmental persistence in soils, vegetation, water, farm environments and food-processing facilities [139,140]. This ecological flexibility, including the capacity for psychrotrophic growth and resilience to dehydration and acidic conditions, suggests that its distribution will be influenced by climate-driven shifts in temperature and moisture patterns. However, although changes in environmental conditions are expected to alter its prevalence, the current body of evidence linking climate change directly to listeriosis trends remains less definitive than the clear, temperature-dependent correlations identified for pathogens, such as *Salmonella* spp. and *Vibrio* spp. [39,49,51].

Climate-sensitive mechanisms for *L. monocytogenes* are likely to operate predominantly through environmental dissemination and changes in contamination pathways rather than through a simple temperature–incidence relationship. Heavy rainfall and flooding can lead to contamination of water sources, from which the pathogen may spread to agricultural fields and food-processing settings. Listeriosis outbreaks have been linked to waterborne contamination following extreme weather events [51]. Environmental adaptations in the agricultural sector have the potential to compromise food safety by facilitating the entry of *L. monocytogenes* into the production chain. Specifically, as water scarcity becomes more frequent, an increasing reliance on untreated or recycled wastewater for crop irrigation provides a direct pathway for this environmental pathogen to contaminate the food supply [39,51]. The role of irrigation water as a potential contamination and exposure pathway is well documented in agricultural settings, where *L. monocytogenes* has been detected in both surface water sources and adjacent agricultural soils, with persistence influenced by seasonal and environmental factors [141,142]. Soil moisture, vegetation cover and farm management may also influence persistence in agricultural environments, which is relevant as contamination can occur before harvest or later through food-processing environments where *L. monocytogenes* may persist and form biofilms [139,143].

Although AMR is not currently a major public-health concern in *L. monocytogenes*, the report of resistant isolates in food, animal and environmental sources warrants continued surveillance, particularly given the limited therapeutic options for invasive listeriosis [9,144,145]. As the zoonosis associated with the highest mortality and severity in the EU, listeriosis poses an emerging threat to high-risk populations, including the geriatric, pregnant, and immunocompromised. Recent surveillance underscores a significant multi-year surge in confirmed infections, culminating in the highest annual number of cases recorded in the EU since systemic reporting began in 2007 [9].

In Portugal, *L. monocytogenes* has been studied across a range of contexts relevant to food safety, clinical surveillance and One Health, generating national evidence without explicit climate orientation (Supplementary Table S1) [74,98,130,145,146,147,148,149,150,151,152,153].

Internationally, the evidence linking climate-related factors to *L. monocytogenes* is markedly weaker and more contested than for the other pathogens covered in this review. A systematic analysis of how climate factors influence *Listeria* spp. highlighted massive research gaps, noting a complete absence of published data on thermal limits, extreme rainfall, or specific temperature thresholds. The review concluded that direct effects of climate change on European listeriosis rates are highly improbable. However, shifts in weather patterns could still increase infections indirectly through alternative exposure pathways [5]. A Canadian five-pathogen surveillance study conducted in Ontario (2010–2017) found that *L. monocytogenes* incidence, although the lowest among the pathogens studied, nonetheless increased during summer months, alongside *Salmonella*, *Yersinia* and STEC [154]. At the food-chain level, a Spanish study of iceberg lettuce production in the southeast of the country assessed *Salmonella* spp., *E. coli*, and *L. monocytogenes* contamination following an extreme flooding event, identifying solar radiation, alongside seasonal temperature, as a critical climatic driver of post-flood pathogen decline in the field [155].

No extreme climatic event has been directly linked to *L. monocytogenes* transmission, persistence or AMR internationally, in Europe or elsewhere, in the literature identified for this review. Given the position that this pathogen’s climate sensitivity operates, if at all, through indirect rather than direct pathways, this absence is broadly consistent with current understanding rather than a straightforward evidence gap, though it nonetheless leaves open whether climate factors could plausibly affect the environmental reservoirs, persistence, transmission and AMR of this species.

Collectively, Portuguese studies on *L. monocytogenes* span clinical surveillance, food-safety monitoring, processing-plant environmental persistence, AMR characterisation and dairy farm ecology, constituting a notably broad national research record for this pathogen. However, studies explicitly connecting *L. monocytogenes* with soils, irrigation water, flooding, drought, climate change or environmental AMR remain limited. The key gap is therefore the integration of the well-developed national food-safety and clinical surveillance evidence base with environmental and climate-sensitive transmission pathways.

### 4.5. Shigella *spp.*

Compared with *Salmonella* spp. and *Vibrio* spp., the relationship between *Shigella* spp. and climate change is less directly ecological and more strongly mediated by water, sanitation and hygiene conditions. A systematic review and meta-analysis of 83 studies found that all analysed reports for *Shigella* spp. showed a positive association with ambient temperature, while 90% of studies reported a positive association with precipitation and flooding, consistent with broader patterns observed for other enteric FW pathogens [49]. Earlier time-series studies from Asia also found associations between bacillary dysentery or shigellosis and climatic variables, including temperature, rainfall and humidity, suggesting that climate variability can influence transmission where water quality and sanitation are vulnerable [156,157]. Recent One Health reviews similarly identify flooding, rainfall variability and disruption of water systems as relevant pathways for climate-sensitive transmission of enteric pathogens, including *Shigella* spp. [37,51].

The mechanisms differ from those described for environmentally persistent bacteria. Humans are the principal reservoir of *Shigella* spp., and transmission occurs mainly through the faecal–oral route, but infections can also be transmitted through contaminated food and water. Climate-related effects are therefore more likely to arise from contamination of drinking water, food-preparation environments and household settings than from expansion of long-term environmental reservoirs [158]. Flooding may increase exposure by damaging sanitation infrastructure and mobilising faecal contamination, whereas drought and water scarcity may reduce hygiene capacity and increase dependence on unsafe water sources [13,37]. Person-to-person transmission is well illustrated by a Portuguese outbreak at a child care institution in Queluz in early 1995, in which introduction of the pathogen into a single centre led to secondary spread affecting 99 children, 17 related household and 7 staff members [159].

AMR is a central and emerging concern for *Shigella* spp. since MDR and extensively drug-resistant (XDR) lineages have emerged internationally, including strains resistant to fluoroquinolones, azithromycin and third-generation cephalosporins [160,161]. While EU/EEA notification rates have returned to pre-pandemic levels following recent multi-country outbreaks, U.S. surveillance shows that XDR isolates surged to 8.5% of reported cases by 2023. These highly resistant strains can result in significant clinical severity, with roughly 38% of U.S. patients requiring hospitalization and occasional fatalities documented in EU foodborne clusters [9,46,162]. In Portugal, however, the evidence base for shigellosis relies largely on European outbreak tracking rather than on dedicated national surveillance programs (Supplementary Table S1) [9,163,164].

Internationally, the temperature sensitivity of *Shigella* spp. has been assessed. A comprehensive systematic review and meta-analysis of 83 publications investigating *Salmonella*, *Shigella*, *Vibrio*, and *Listeria* species confirmed that warmer ambient temperatures consistently correlate with higher rates of gastroenteritis. Furthermore, the majority of the reviewed literature identified a positive relationship between precipitation levels and increased enteric infections [49]. In Belgium, a province-level analysis integrating weekly meteorological data with epidemiological records demonstrated that periods of very high temperatures correlated with elevated incidence rates for several key pathogens. Specifically, these warm-weather anomalies were associated with increased cases of *E. coli*, *Salmonella* spp., and *Shigella* spp., as well as parasitic gastroenteritis and *Borrelia burgdorferi* infections [165]. A Greek study comparing four *S. sonnei* waterborne outbreaks recorded over three decades explicitly examined their association with environmental, seasonal and climatological conditions. The study found attack rates of 9–13% and identified two distinct epidemic-wave patterns corresponding to different environmental conditions. It also noted that similar waterborne *Shigella* outbreaks have been reported in other Mediterranean countries, including Spain and Israel [166]. In Oregon, USA, a *S. sonnei* outbreak during the historically wet 2015–2016 rainy season revealed a 36% increase in shigellosis risk per inch of precipitation specifically among individuals experiencing homelessness, while no such association was observed in the housed population. This finding demonstrated that climate-sensitive enteric pathogen transmission may be strongly mediated by housing vulnerability, sanitation access and other social determinants, rather than by climate exposure alone [167].

In Portugal, *Shigella* spp. appears to be studied primarily through clinical diagnosis, outbreak investigation and AMR surveillance, rather than through food, soil, wastewater or climate-oriented environmental monitoring. The main gap is therefore the absence of integrated studies assessing whether extreme precipitation, flooding, drought, wastewater contamination or water scarcity may influence *Shigella* spp. transmission risk in mainland Portugal.

### 4.6. Yersinia enterocolitica

Compared with other pathogens discussed in this review, *Y. enterocolitica* has received considerably less attention in climate change research. Current evidence linking climatic variables to yersiniosis is limited, and most epidemiological studies have focused on seasonal patterns rather than on long-term climate change or extreme weather events [9,168]. Notably, yersiniosis shows substantially less seasonal variation than other zoonotic enteric diseases with its temporal distribution remaining relatively constant throughout the year and only minor increases observed in June, July, and September. This pattern suggest that yersiniosis may be driven more strongly by relatively stable year-round transmission pathways than by fluctuating environmental or climatic factors [168].

*Y. enterocolitica* is psychrotrophic and can survive and replicate at refrigeration temperatures, making food storage practices and cold-chain management particularly important for transmission [169]. Domestic pigs are recognised as the principal reservoir of pathogenic strains, being frequently detected in pig tonsils and intestines and capable of contaminate carcasses and pork products during slaughter. Bioserotype 4/O:3 is the most common cause of human illness in continental Europe [170]. Human infections are primarily associated with pork products, although *Y. enterocolitica* has also been detected in environmental sources such as water and wildlife [9,170]. In recent years, an increasing number of yersiniosis events have also been linked to fresh produce, in addition to pork, broadening the food-chain exposure pathways relevant to surveillance [170]. Climate-related impacts are therefore likely to occur indirectly through changes in livestock production systems, water quality, agricultural runoff and environmental contamination, rather than solely through direct temperature-driven proliferation [39]. Environmental persistence may also be relevant, as *Y. enterocolitica* can survive in soil and natural waters, with persistence varying by temperature and serotype and generally favouring longer survival at lower temperatures [171,172].

AMR in *Y. enterocolitica* remains less comprehensively characterised than in other Enterobacterales. Nevertheless, resistant isolates have been reported from human, animal and food sources, and MDR strains have been detected in pig tonsils across different production systems, with resistance patterns linked to farm type and production intensity [173].

The prevalence and geographic distribution of *Y. enterocolitica* in swine and wild boar populations appear to be influenced by specific environmental variables, showing a positive correlation with annual precipitation and a negative correlation with average air temperatures. Studies from regions such as China and Northern Spain showed that the highest infection rates are found in mountainous or colder areas characterized by high rainfall [170]. However, prevalence has also been reported during warmer months, including a higher prevalence in intestinal samples collected in Finland during July and August, a 13.7% pig prevalence in France during warm periods, and summer human outbreaks around the world [170]. Whether climate-related environmental changes contribute to the dissemination of resistant *Y. enterocolitica* populations remains largely unknown.

No extreme climatic event, such as a specific flood, heatwave or drought, has been directly linked to *Y. enterocolitica* transmission, persistence or AMR in the international literature identified for this review. This absence itself is notable given that flooding and extreme rainfall have been linked to the transmission of other FW pathogens considered in this review.

In Portugal, a comprehensive One Health review of *Y. enterocolitica* in the pork production chain was published, synthesizing global data on its epidemiology, virulence, and food safety risks (Supplementary Table S1). The authors identify the One Health approach as essential for managing the pathogen and emphasize the urgent need for enhanced farm-to-fork surveillance. Notably, the review implicitly highlights a major knowledge gap. While Portugal participates in European surveillance through EFSA and ECDC, the authors identified no primary Portuguese studies regarding the pathogen’s prevalence in livestock, the environment, or the specific influence of climate variability on its transmission within the country [170].

## 5. Discussion: Knowledge Gaps and Research Priorities in Portugal

Overall, the literature indicates that climatic factors may modify the environmental persistence, dissemination and exposure pathways of FW bacterial pathogens, although the evidence varies considerably among agents. International evidence is strongest and most direct for *Vibrio* spp. and comparatively extensive for *Salmonella* spp. Evidence for pathogenic *E. coli* is moderate, particularly regarding temperature, rainfall and hydrological events. Evidence for *Shigella* spp. is less extensive and strongly influenced by water, sanitation and hygiene conditions, whereas climate-related findings for *L. monocytogenes* and *Y. enterocolitica* remain limited or largely indirect. In Portugal, evidence is comparatively more developed for *Salmonella* spp. and *Vibrio* spp., substantial but compartmentalised for pathogenic *E. coli* and *L. monocytogenes*, limited for *Shigella* spp., and especially scarce for *Y. enterocolitica*. These comparisons do not imply equivalent evidentiary strength or public-health relevance. However, Portuguese studies remain poorly integrated with climate science and climate-oriented environmental surveillance remains limited or absent, preventing conclusions about whether, or to what extent, climatic factors may have influenced pathogen transmission or AMR dissemination in mainland Portugal (Supplementary Table S1).

This review has some limitations that should be considered when interpreting its findings. Its narrative rather than systematic design, with reliance primarily on PubMed, may have introduced selection bias and limited the reproducibility and comprehensiveness of the evidence synthesis. Studies published in both English and Portuguese were considered, and selected reports were consulted for contextual information. Nevertheless, relevant publications not indexed in PubMed, as well as technical and surveillance reports, thesis and other literature, may have been overlooked. Publication bias may also have favoured studies reporting statistically significant associations between environmental variables and microbial outcomes. The predefined pathogen panel was aligned with the objectives of a subsequent research phase and the established scope and expertise of the laboratory, meaning the exclusion of *Campylobacter* spp. and other FW pathogens which limits the scope of the review. Considerable heterogeneity was observed across study designs, sampling strategies, environmental matrices, geographic settings, analytical methods and reported outcomes, limiting direct comparisons and preventing quantitative synthesis. The available evidence was also unevenly distributed among pathogens and environmental compartments, with particularly limited climate-oriented research from Portugal. Apparent evidence gaps may also reflect surveillance bias arising from differences in mandatory notification, diagnostic testing, laboratory capacity and environmental monitoring rather than true differences in pathogen occurrence. A consistent structure was nevertheless maintained across all six pathogens to facilitate comparison and highlight these differences, rather than to imply equivalent evidentiary strength or public-health relevance. Finally, short-term meteorological associations cannot be directly attributed to long-term climate change, particularly for AMR, for which antimicrobial use remains the principal driver and climate-related factors may act mainly by modifying environmental persistence, dissemination and exposure pathways.

In light of these findings and the methodological limitations described above, a major priority is therefore the development of climate-informed One Health surveillance, which integrates pathogen and AMR monitoring across human, animal and environmental compartments with climatic data, rather than monitoring microbial occurrence and resistance alone (Figure 2). Future studies should integrate environmental sampling from freshwater, wastewater, agricultural soils, sediments, estuaries and coastal waters with antimicrobial susceptibility testing, WGS and environmental metadata, such as temperature, precipitation, salinity, pH, hydrological variability, land and water use, and documented drought and flood events. This approach is particularly relevant for mainland Portugal were drought, water scarcity, extreme precipitation and coastal warming may alter exposure pathways through irrigation, recreational waters, shellfish production and wastewater reuse.

Research should also move beyond pathogen detection alone. Longitudinal studies are needed to assess seasonality, extreme weather events and delayed effects after flooding or drought. Integrating microbiological data with hydrological, meteorological and land-use information would support risk modelling and help identify priority locations for surveillance. In this context, mainland Portugal could serve as a valuable Mediterranean case study for understanding how climate-sensitive environmental reservoirs contribute to the transmission of FW pathogens and the emergence and dissemination of antimicrobial resistance.

## 6. Conclusions

Climate-related changes may influence the occurrence, dissemination and exposure, as well as pathways involved in the environmental spread of ARB, although the strength and mechanisms of these relationships vary among pathogens. Internationally, the evidence suggesting this influence is strongest for *Vibrio* spp., *Salmonella* spp. and pathogenic *E. coli*, while climate-related effects on *Shigella* spp., *L. monocytogenes* and *Y. enterocolitica* remain more indirect or less established. The available Portuguese evidence remains insufficient to determine whether, or to what extent, these factors influence FW pathogen transmission or AMR patterns in Portugal. At a national level there is relevant clinical, food-chain, animal, wildlife and environmental evidence, but the integration of microbiological findings with climatic, hydrological and land-use information remains limited. Climate-informed One Health surveillance should therefore prioritise *Salmonella* spp., pathogenic *E. coli* and *Vibrio* spp. while maintaining targeted surveillance of the remaining pathogens according to public-health relevance and local exposure pathways. Longitudinal and event-based sampling, genomic analysis and predictive models are needed to identify high-risk locations and periods. Such approaches could strengthen preparedness for climate-sensitive microbial risks without attributing AMR directly to climate change.

## Figures and Tables

**Figure 1:**
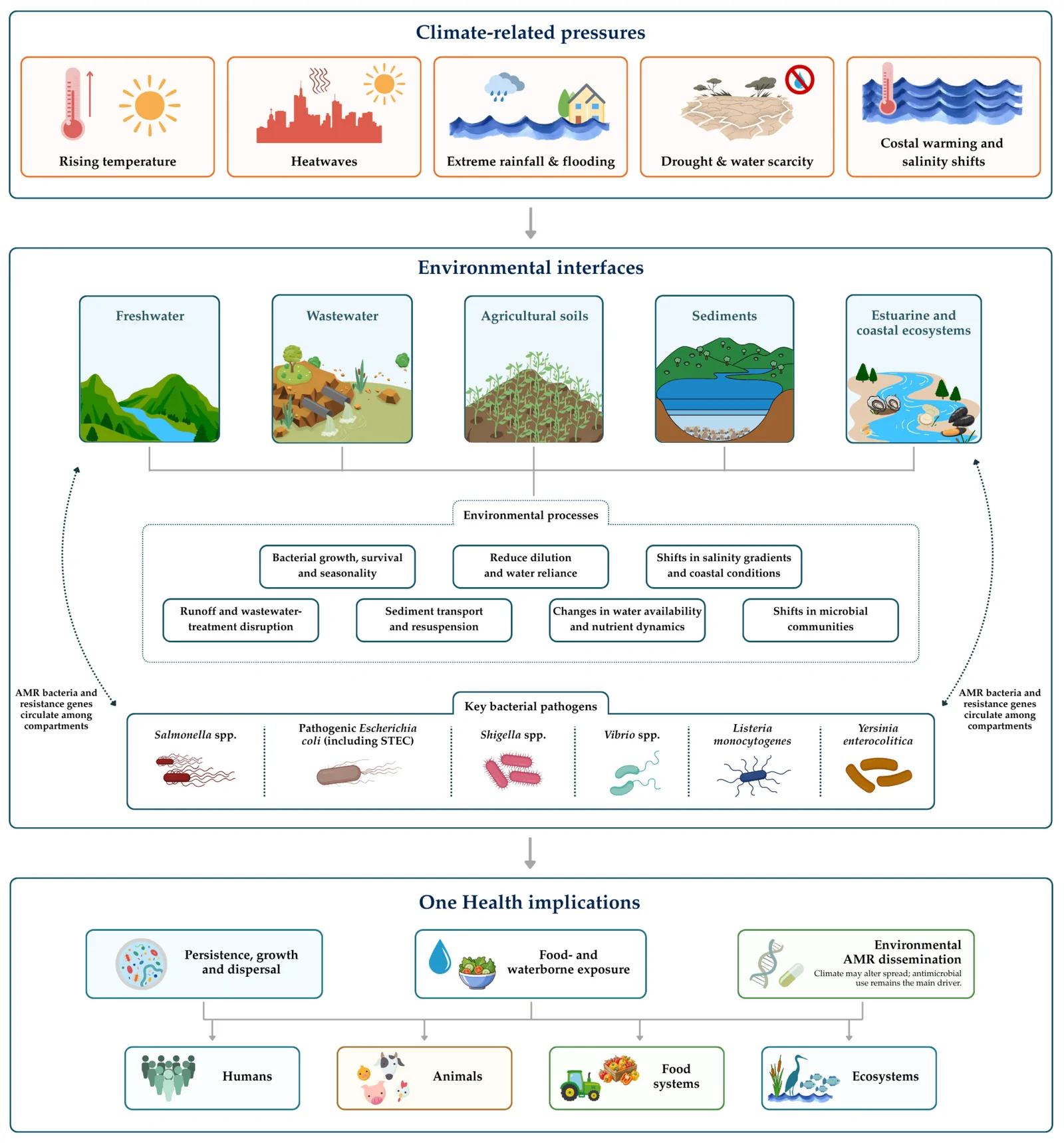
Conceptual One Health framework illustrating how climate-related pressures may modify environmental processes influencing the persistence and dispersal of FW bacterial pathogens, human exposure pathways, and the dissemination of ARB and ARGs. Created with Canva.

**Figure 2:**
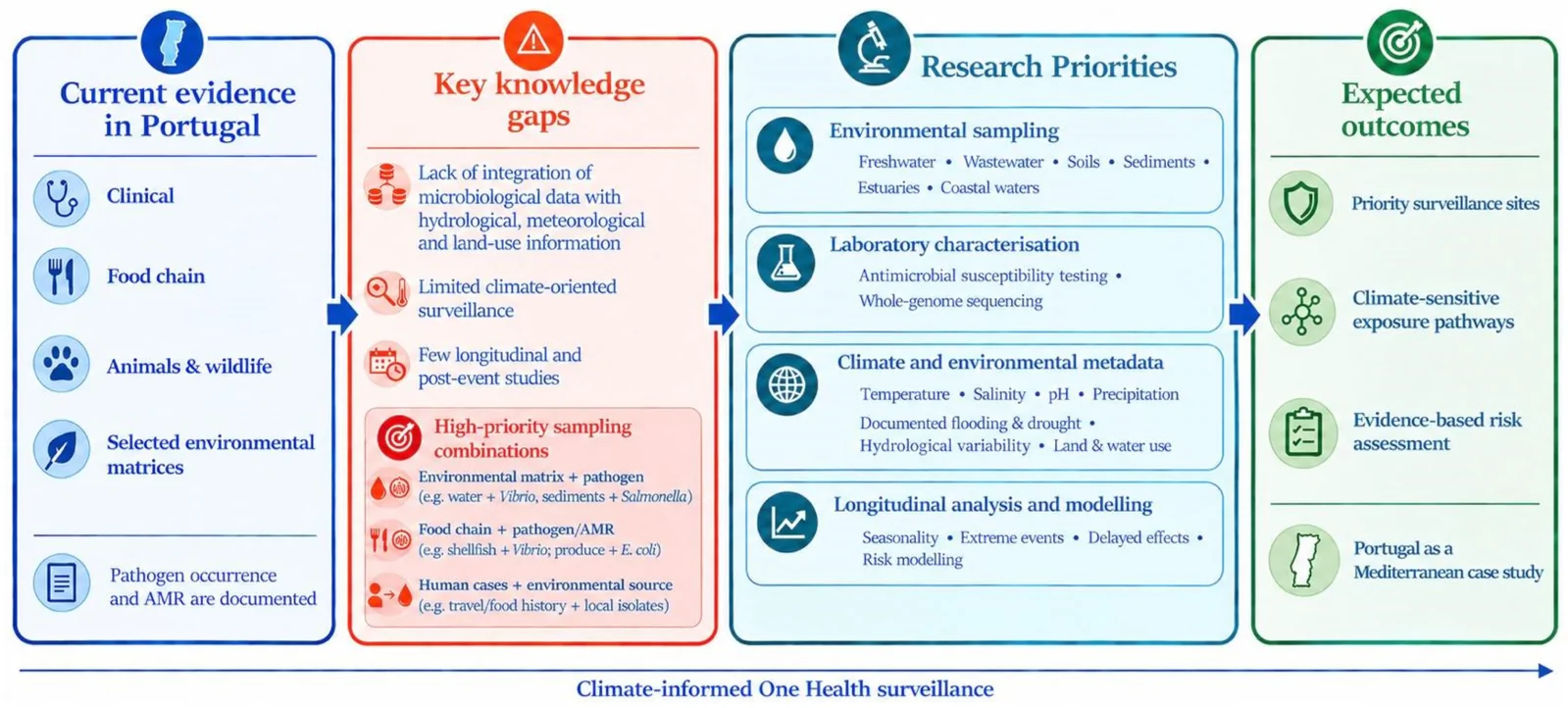
Knowledge gaps and research priorities for climate-informed One Health surveillance of FW bacterial pathogens and AMR in Portugal. Created with Canva.

## Data Availability

No new data were created or analyzed in this study. Data sharing is not applicable to this article.

## Supplementary Materials

The following supporting information can be downloaded at: https://www.mdpi.com/article/10.3390/microorganisms14102231/s1, Supplementary Data S1: Reference search parameters; Supplementary Table S1: Summary of Portuguese studies on FW bacterial pathogens cited in this review, organised by pathogen. Columns indicate study type, matrix or setting studied, availability of AMR and WGS data, and whether climate or environmental metadata were included.
