# Supplementary material for: Food- and Waterborne Bacteria Transmission and Antimicrobial Resistance Under Climate Change: Evidence Gaps in Mainland Portugal
Source: Microorganisms. 2026 Oct 3;14(10):2231. doi: 10.3390/microorganisms14102231 (PMC13707665; doi:10.3390/microorganisms14102231)
Supplement: Supplementary file 1 [file microorganisms-14-02231-s001.zip › Supplementary Data S1.pdf]

Supplementary Data S1: Reference search parameters

The principal bibliographic database consulted for this narrative review was PubMed. Searches were conducted iteratively from database inception to 28/08/2026, with no lower publication-date restriction. The search strategy was refined as relevant literature was identified, and reference lists of selected publications were also screened to identify additional relevant studies.

Searches were tailored to each pathogen and thematic section. The general Boolean structure was: ("Salmonella" OR "Salmonella enterica" OR "Escherichia coli" OR "STEC" OR "VTEC" OR "Shigella" OR "Vibrio" OR "Listeria monocytogenes" OR "Yersinia enterocolitica") AND ("climate change" OR "climate" OR "temperature" OR "warming" OR "ambient temperature" OR "precipitation" OR "rainfall" OR "flood*" OR "drought" OR "water scarcity" OR "salinity" OR "extreme weather" OR "hydrological variability") AND ("persistence" OR "survival" OR "transmission" OR "dissemination" OR "occurrence" OR "incidence" OR "seasonality" OR "ecology" OR "antimicrobial resistance" OR "AMR" OR "antimicrobial resistance genes" OR "ARGs" OR "resistome" OR "wastewater" OR "freshwater" OR "river*" OR "soil*" OR "sediment*" OR "estuar*" OR "coast*" OR "irrigation" OR "water reuse"). Equivalent pathogen-specific searches were conducted by replacing the first block with the name and relevant synonyms of each pathogen. Searches addressing the Portuguese evidence base additionally included ("Portug*"). Broader national searches used the structure: (pathogen-specific terms) AND ("Portug*") AND ("clinical" OR "human" OR "food" OR "food chain" OR "animal" OR "livestock" OR "wildlife" OR "environment*" OR "water" OR "wastewater" OR "soil*" OR "sediment*" OR "antimicrobial resistance" OR "AMR" OR "whole-genome sequencing" OR "WGS"). Search terms were adapted when necessary to examine specific climatic factors, environmental reservoirs, transmission pathways or AMR outcomes. Reference lists of relevant reviews, primary studies and outbreak reports were also examined to identify additional publications.

Eligible sources included publications in English or Portuguese addressing at least one of the six selected bacterial pathogens in relation to climatic or environmental factors, occurrence, persistence, survival, transmission, seasonality, environmental reservoirs or AMR. Experimental, observational, epidemiological, surveillance and environmental-monitoring studies were considered. Portuguese studies conducted in clinical, food-chain, animal, wildlife or environmental settings were eligible when they directly addressed the review objectives or contributed relevant national context. International studies were included when they provided mechanistic, epidemiological, comparative or methodological evidence relevant to the climate–pathogen–AMR framework. Peer-reviewed review articles were used to frame major topics and identify additional literature, while primary studies were used to support specific findings. Authoritative surveillance reports, technical documents, regulatory publications and reports from national or international organisations were also consulted when required to describe epidemiological, climatic, environmental, surveillance or regulatory contexts. These documents were used primarily for contextual information rather than as evidence of causal relationships.

Duplicate records, conference abstracts, preprints, editorials, commentaries, unpublished theses, non-authoritative literature and publications in languages other than English or Portuguese were excluded. Studies that did not address the selected pathogens or contribute to the climatic,

environmental, transmission or AMR dimensions of the review were also excluded. Because this was a targeted narrative review, the Portuguese literature was not intended to constitute an exhaustive inventory of every study involving the selected pathogens. Studies directly aligned with the objectives of the review were considered, whereas the broader body of clinical or experimental research without a direct connection to climatic factors, environmental pathways or AMR was not comprehensively included. In these areas, selected studies were used to provide a representative overview, prioritising those with the greatest relevance to the review objectives, national surveillance, public-health context or environmental transmission pathways. Given the breadth and heterogeneity of the available evidence, findings were integrated through narrative synthesis within a One Health framework rather than through a formal systematic-review process.
