# Supplementary material for: Food- and Waterborne Bacteria Transmission and Antimicrobial Resistance Under Climate Change: Evidence Gaps in Mainland Portugal
Source: Microorganisms. 2026 Oct 3;14(10):2231. doi: 10.3390/microorganisms14102231 (PMC13707665; doi:10.3390/microorganisms14102231)
Supplement: Supplementary file 1 [file microorganisms-14-02231-s001.zip › Supplementary Table S1.pdf]

Supplementary Table S1. Summary of Portuguese studies on FW bacterial pathogens cited in this review, organised by pathogen. Columns indicate study type, matrix or setting studied, availability of AMR and WGS data, and whether climate or environmental metadata were included.

Pathogen	Reference	Study Type	Matrix / Setting	Climate / Env. Metadata
Salmonella spp.	Pista *et al*. (2022) [22]	Wildlife	Wild animals, 2 conservation centres	No
	Batista *et al*. (2023) [23]	Wildlife	Free-living birds, mainland Portugal	No
	Antunes *et al*. (2016) [54]	Review	Poultry meat (global, PT authors)	No
	Campos *et al*. (2019) [55]	Review	Pig production chain (global, PT authors)	No
	Silveira *et al*. (2021) [56]	Import monitoring	S. Heidelberg/Minnesota, imported poultry	No
	Alba *et al*. (2026) [57]	AMR/genomic	S. Infantis, pESI megaplasmid, EU-wide	No
	Gomes-Neves *et al*. (2012) [58]	Food-producing animals	8 abattoirs, pigs & meat handlers	No
	Cota *et al*. (2019) [59]	Food-producing animals	120 pigs, single abattoir	No
	Moura-Alves *et al*. (2022) [60]	Food-producing animals	Swine carcasses, 2 slaughterhouses N-PT	No
	Ribeiro-Almeida *et al*. (2024) [61]	Companion animal	Raw meat-based pet diets	No
	Correia *et al*. (2004) [62]	Outbreak surveillance	27 outbreaks, N Portugal, 2002	No
	McGeoch *et al*. (2024) [63]	Outbreak/genomic	S. Saintpaul, cantaloupe, UK/PT	No
	Freixo *et al*. (2011) [64]	Clinical	S. Typhimurium, children, 2 hospitals	No
	von Schreeb *et al*. (2026) [65]	Outbreak/genomic	S. Typhimurium, fresh cheese	No
	Antunes *et al*. (2018) [66]	Aquaculture/environmental	Rainbow trout farms & retail trout	No
	Rodrigues *et al*. (2024) [67]	Wildlife/environmental	Gulls, Porto coastline, 2008 & 2023	No
	Cota *et al*. (2021) [68]	Companion animal	Pet reptiles	No
	Pereira *et al*. (2025) [69]	Food-producing animals	Small ruminants, 122 farms	No
	Miranda *et al*. (2023) [70]	Food-producing animals	Indigenous layer hen breeds	No
	Catalão Dionísio *et al*. (2000) [71]	Environmental/estuarine	Coastal & estuarine waters	No
	Mansilha *et al*. (2010) [72]	Environmental/bathing water	EU-compliant recreational beaches	No
	Rodrigues *et al*. (2016) [73]	Environmental/methodology	Transitional & coastal waters	No
	Mendonça *et al*. (2024) [74]	Food safety	Raw milk cheese, Beira Baixa	No
	Antunes *et al*. (2011) [75]	Clinical/AMR	S. Enteritidis ST11, qnrS1 plasmid	No
	Seixas *et al*. (2016) [76]	National surveillance/AMR	S. 1,4,[5],12:i:- ASSuT, 2006–2011	No
	Campos *et al*. (2016) [77]	AMR	National collection, mcr-1, 2011–2015	No
	Silveira & Pista (2023) [78]	Clinical/genomic	S. Typhimurium, mcr-9	No
	Leão *et al*. (2024) [79]	Clinical/genomic	S. Enteritidis, human/non-human	No

Pathogen	Reference	Study Type	Matrix / Setting	Climate / Env. Metadata
Salmonella spp.	Costa *et al*. (2022) [80]	National surveillance/AMR	Food-borne pathogens, food & animals, 2014–2019	No
	Gonçalves *et al*. (2024) [81]	Food-producing animals	Pigs at slaughterhouse & pork meat	No
**Pathogenic** *Escherichia coli*	Pista *et al*. (2022) [22]	Wildlife	Wild animals, 2 conservation centres	No
	Batista *et al*. (2023) [23]	Wildlife	Free-living birds, mainland Portugal	No
	Freire *et al*. (2022) [34]	Wildlife/AMR	Urban pigeons, Lisbon	No
	Aires-de-Sousa *et al*. (2020) [35]	Wildlife/AMR	Gull faeces, Lisbon coastline	No
	Gomes *et al*. (2024) [36]	Environmental/recreational	Recreational waters, NW Portugal	No
	Ribeiro-Almeida *et al*. (2024) [61]	Companion animal	Raw meat-based pet diets	No
	Pereira *et al*. (2025) [69]	Food-producing animals	Small ruminants, 122 farms	No
	Mendonça *et al*. (2024) [74]	Food safety	Raw milk cheese, Beira Baixa	No
	Costa *et al*. (2022) [80]	National surveillance/AMR	Food-borne pathogens, food & animals, 2014–2019	No
	Gonçalves *et al*. (2024) [81]	Food-producing animals	Pigs at slaughterhouse & pork meat	No
	Dias *et al*. (2022) [97]	Wildlife	Wild boar, deer, fox, otter (STEC)	No
	Praça *et al*. (2023) [98]	Food safety	Cured raw milk cheese, Alentejo	No
	Varela *et al*. (2015a) [99]	Environmental/clinical	Gulls, wastewater, effluent, patients	No
	Santos *et al*. (2025) [100]	Wildlife rehabilitation	Storks, egrets, hedgehogs	No
	Bessa *et al*. (2014) [101]	Environmental/river	River water, upstream/downstream WWTP	No
	Oliveira *et al*. (2016) [102]	Environmental/wastewater	14 municipal + 8 poultry WWTPs	No
	Varela *et al*. (2015b) [103]	Environmental	Urban streams & effluents	No
	Freire *et al*. (2023) [104]	Environmental/marine	Bivalves, shellfish production areas	No
	Quinteira *et al*. (2024) [105]	Food-producing animals	Dairy & autochthonous cattle, N Portugal	No
	López-Lorenzo *et al*. (2025) [107]	Food-producing animals (cross-border)	Pig farms, NW Iberian Peninsula	No
	Ribeiro-Almeida *et al*. (2025) [108]	Food-producing animals	Rabbit meat production	No
	Menezes *et al*. (2026) [109]	Companion animal/genomic	Human-companion animal interface	No
	Ballem *et al*. (2021) [96]	Food-producing animals	Dairy cattle, N Portugal (STEC)	No
	Cravo *et al*. (2025) [110]	Environmental/bivalves	Ria Formosa water quality index	Yes
	Caetano *et al*. (2023) [111]	Environmental/estuarine	Ria Formosa lagoon, treated effluents	Yes
Vibrio spp.	Blake *et al*. (1977a) [127]	Outbreak/historical	Cholera epidemic, 1974, national	No
	Blake *et al*. (1977b) [128]	Outbreak/historical	Cholera epidemic, bottled water	No
	Albuquerque *et al*. (2013) [129]	Clinical	V. cholerae non-O1/non-O139 bacteraemia	No
	Pereira *et al*. (2001) [130]	Food safety	Molluscan shellfish, Portugal	Yes

Pathogen	Reference	Study Type	Matrix / Setting	Climate / Env. Metadata
Vibrio spp.	Guedes *et al*. (2023) [131]	Food safety	Ready-to-eat Pacific oysters	No
	Anacleto *et al*. (2013a) [132]	Environmental/estuarine	Clams & sediment, Tagus estuary	Yes
	Rodrigues *et al*. (2023) [133]	Environmental/marine	Pacific oysters, 1-year survey	Yes
Listeria monocytogenes	Mendonça *et al*. (2024) [74]	Food safety	Raw milk cheese, Beira Baixa	No
	Praça *et al*. (2023) [98]	Food safety	Cured raw milk cheese, Alentejo	No
	Pereira *et al*. (2001) [130]	Food safety	Molluscan shellfish, Portugal	No
	Barbosa *et al*. (2013) [145]	AMR	Food & clinical isolates, 2003–2007	No
	Chambel *et al*. (2007) [146]	Food processing	Ewe & cow cheese dairies	No
	Almeida *et al*. (2013) [147]	Food processing	3 cheese-processing plants	No
	Nunes *et al*. (2024) [148]	Farm	Dairy farms (feed, water, faeces)	No
	Almeida *et al*. (2006) [149]	Clinical/epidemiology	Hospital records, 1994–2003	No
	Almeida *et al*. (2010) [150]	Clinical	Clinical isolates, 1994–2007	No
	Magalhães *et al*. (2014) [151]	Clinical	Clinical isolates, 2008–2012	No
	Magalhães *et al*. (2015) [152]	Clinical/outbreak	Cheese outbreak, Alentejo, 2009–2012	No
	Ferreira *et al*. (2018) [153]	WGS	Outbreak strain genome sequence	No
Shigella spp.	Suspiro & Menezes (1996) [159]	Outbreak	Child care institution, Queluz, 1995	No
	ECDC/UKHSA outbreak report (2023) [164]	Outbreak surveillance	S. sonnei, Cabo Verde travel-associated	No
Yersinia enterocolitica	Saraiva *et al*. (2025) [170]	Review (One Health)	Swine production chain (review)	No
